# Multiscale Computational Model Reveals Nerve Response in a Mouse Model for Temporal Interference Brain Stimulation

**DOI:** 10.3389/fnins.2021.684465

**Published:** 2021-06-30

**Authors:** Jose Gomez-Tames, Akihiro Asai, Akimasa Hirata

**Affiliations:** ^1^Department of Electrical and Mechanical Engineering, Nagoya Institute of Technology, Nagoya, Japan; ^2^Center of Biomedical Physics and Information Technology, Nagoya Institute of Technology, Nagoya, Japan

**Keywords:** transcranial temporal interference stimulation, brain stimulation, multiscale model, mouse model, neural model, envelope

## Abstract

There has been a growing interest in the non-invasive stimulation of specific brain tissues, while reducing unintended stimulation in surrounding regions, for the medical treatment of brain disorders. Traditional methods for non-invasive brain stimulation, such as transcranial direct current stimulation (tDCS) or transcranial magnetic stimulation (TMS), can stimulate brain regions, but they also simultaneously stimulate the brain and non-brain regions that lie between the target and the stimulation site of the source. Temporal interference (TI) stimulation has been suggested to selectively stimulate brain regions by superposing two alternating currents with slightly different frequencies injected through electrodes attached to the scalp. Previous studies have reported promising results for TI applied to the motor area in mice, but the mechanisms are yet to be clarified. As computational techniques can help reveal different aspects of TI, in this study, we computationally investigated TI stimulation using a multiscale model that computes the generated interference current pattern effects in a neural cortical model of a mouse head. The results indicated that the threshold increased with the carrier frequency and that the beat frequency did not influence the threshold. It was also found that the intensity ratio between the alternating currents changed the location of the responding nerve, which is in agreement with previous experiments. Moreover, particular characteristics of the envelope were investigated to predict the stimulation region intuitively. It was found that regions with high modulation depth (| maximum| − | minimum| values of the envelope) and low minimum envelope (near zero) corresponded with the activation region obtained via neural computation.

## Introduction

There has been increasing interest in the non-invasive electrostimulation of specific parts of the brain. Recently, as one of the topics in this field, stimulation of the deep brain region has gained attention (DaSilva et al., 2015; Csifcsák et al., 2018; Gomez-Tames et al., 2019a, 2020a; Huang and Parra, 2019; Bikson and Dmochowski, 2020). In conventional non-invasive stimulation techniques, such as transcranial electrical stimulation [e.g., transcranial alternating current stimulation (tACS); transcranial direct current stimulation (tDCS)] and transcranial magnetic stimulation (TMS), can stimulate specific brain regions, but they also simultaneously stimulate the surrounding brain and non-brain regions (e.g., nociceptive fibers stimulation in the scalp) that lie between the stimulator location and target area (Chib et al., 2013; Weber et al., 2014; Huang et al., 2017; Bikson and Dmochowski, 2020; Gomez-Tames et al., 2020a, 2021).

Temporal interference (TI) stimulation has attracted significant attention as it may achieve stimulation of specific cortical or deep brain regions without activation of superficial parts (Laakso and Hirata, 2013; Vossen et al., 2015). TI makes use of two sets of tACS, whose injection current frequencies slightly differ from each other, that may cause a beat wave at a specific area of the brain, enabling position-selective stimulation (Grossman et al., 2017). This stimulation method has been conventionally applied to peripheral stimulation, such as sacral nerve stimulation (Johnson and Tabasam, 2003; Beatti et al., 2011). TI can be considered for stimulation due to the low-pass filtering effect of the passive cell membrane that may be accompanied by rectification of the ionic part (Middleton et al., 2006; Grossman et al., 2017; Mirzakhalili et al., 2020). If a current (a few kHz up to 10 kHz) is injected, the cell membrane in the brain may not readily follow the oscillation of the electric field. Instead, a small difference in the frequencies of the two injection currents may generate a beat wave (modulation envelope) causing neural stimulation. Previous studies have shown promising results for motor area stimulation in mice (Grossman et al., 2017; Song et al., 2020). In addition, various efforts have been made to clarify the mechanism regarding biophysics using neural models (Karimi et al., 2019; Cao and Grover, 2020; Howell and McIntyre, 2020; Mirzakhalili et al., 2020; Esmaeilpour et al., 2021). It is also relevant to incorporate and validate the effects of the interferential currents that are shaped by the anatomical and electrical properties of biological tissues (Rampersad et al., 2019).

In the non-invasive brain stimulation studies, the electric current, as a physical agent, is computed using a volume conductor model, in which the human head (not only the brain) is considered as an inhomogeneous conductor without considering the neural model (Datta et al., 2009; Bikson et al., 2013; Laakso et al., 2015; Opitz et al., 2015; Antonenko et al., 2019; Kasten et al., 2019). Here, the electric current is shaped by the electrical and anatomical characteristics of the head. However, the multiscale modeling, or the interaction with the axon, is needed to replicate the neuromodulation effects in the computational domain (Wongsarnpigoon and Grill, 2008; Salvador et al., 2011; Gomez-Tames et al., 2019b; Seo and Jun, 2019). Several studies have been conducted for multiscale modeling, particularly for TMS (De Geeter et al., 2015; Goodwin and Butson, 2015; Gomez-Tames et al., 2019c), where the pulse frequency is of the order of kHz that have been able to predict experimental thresholds (Gomez-Tames et al., 2018, 2020b; Aberra et al., 2020). If the stimulation of the mouse cortex can be explained in terms of multiscale modeling, the abovementioned hypothesis can be clarified, except for the possible synaptic effect (Gomez-Tames et al., 2019b; Neudorfer et al., 2021).

Here, we present multiscale computational modeling to explore the effects of interferential stimulation on the mouse motor cortex for first time based on experimental measurements. In addition, the characteristics of the generated envelope were explored to intuitively predict the stimulation region.

## Model and Methods

### Mouse Model

**FIGURE 1:**
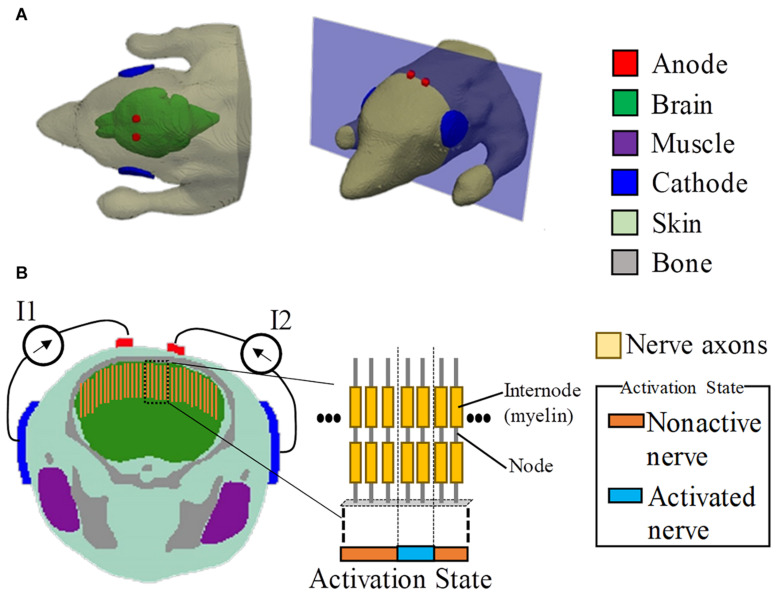
Multiscale model for interferential transcranial alternate current stimulation. **(A)** Volume conductor of the mouse model with two pairs of electrodes attached on the scalp based on the experimental scenario in Grossman et al. (2017). **(B)** Cross-section showing the positions of the axonal nerves in the motor area of the brain (total number of test axons is 80). The activated nerves are depicted by an activation state region (1D).

The mouse model used in this study was developed using computer tomography (Dogdas et al., 2007). The model has the resolution of 0.1 mm and comprises 21 tissues. The dimensions of the model are 38.0 mm × 99.2 mm × 20.8 mm, excluding the tail. In this study, the model was truncated around the neck region because the remaining body part did not influence the current flow in the brain. The head model includes six tissues, namely, skin, brain, muscle, bone, eye, and glands, as shown in Figure 1. Owing to the limitation of image resolution, the thickness of the cerebrospinal fluid is not well modeled.

### Volume Conductor Model

The electric potential generated by the current injected from the electrodes attached to the rat scalp was computed using the scalar potential finite-difference method, with successive over-relaxation and multigrid methods (Dawson and Stuchly, 1998; Laakso and Hirata, 2012) to solve the scalar potential equation:

(1) $$\nabla \cdot \left(\sigma \,\nabla V_{e}\right)=0,$$

where *V*_*e*_ and σ denote the scalar potential and tissue conductivity, respectively. Then, the electric field was obtained by dividing the potential between the two nodes along the edge of a cubic voxel (the minimum component of the model) by the length of the voxel edge. We assigned the electrical conductivity of tissue to each tissue based on the fourth order Cole–Cole model at 1 kHz and literature values (Gabriel et al., 1996; Bernabei et al., 2014). The electrical conductivity did not change significantly in the lower kHz range; thus, the value of 1 kHz is used throughout this study. The electrical conductivities of the skin, brain, muscle, bone, eye, and glands were 0.1, 0.33, 0.321, 0.02, 1.5, and 0.67 S/m, respectively.

### Axon Model

The effects of the extracellular electric field on nerve axons were described by the following general equation (McNeal, 1976; Rattay, 1999):

(2) $$c_{m,n}\,\frac{d\,V_{m,n}}{d\,t}+I_{i\,o\,n,n}-\frac{V_{m,n-1}-2V_{m,n}+-2V_{m,n+1}}{R}=\frac{V_{e,n-1}-2V_{e,n}+-2V_{e,n+1}}{R},$$

where *c*_*m,n*_ is the membrane capacitance, and *V*_*m,n*_ is the membrane potential at position *n*. The interferential current *V*_*e,n*_ was obtained using the scalar potential finite-difference, and the axon of a myelinated nerve consists of internodes (segments ensheathed by myelin) and nodes of Ranvier (ionic channels). The variable *R = 0.5* × (*R*_*n*_+*R*_*n*+1_) denotes the intra-axonal resistance between the centers of two adjacent compartments (nodes and internodes). At the myelinated internodes, the leak conductance was modeled as a passive element. At the nodes of Ranvier, the ionic membrane current was formulated as a conductance-based voltage-gated model based on the Chiu–Ritchie–Rogart–Stagg–Sweeney model (Sweeney et al., 1987), which has been able to reproduce experimental results for motor stimulation of the brain cortex (Gomez-Tames et al., 2018, 2019b). The model parameters are presented in Table 1. The length and diameter of an axon in a mouse have been reported to be 2 mm and 1 μm, respectively (Ong et al., 2009). No measured results have been reported for the myelin thickness. Thus, the original parameters *C*_*m,in*_ and *C*_*m,n*_ were increased linearly by a factor of 15, considering that the thickness of the myelin reduces with the ratio of axon thickness of a human to that of a mouse (Arancibia-Cárcamo et al., 2017). No adjustments were applied for fine-tuning. Due to the lack of detailed anatomy of the mouse brain, we simply located the axons perpendicular to the brain cortex, as shown in Figure 1.

**TABLE 1:** Parameters of the modified CRRSS model.

Parameter	Value
Nernst potential for sodium channels (*V*_*Na*_)	115 mV
Nernst potential for leakage channels (*V*_*l*_)	−0.01 mV
Capacity of membrane at internode (*C*_*m*_)	402 nF
Capacity of membrane at node (*C*_*m*_)	452 nF
Internode membrane resistivity (*R*_*m*_)	219 kΩ
Nodal membrane resistivity (*R*_*m*_)	3.26 kΩ
Myeline conductance (*G*_*m*_)	26.8 nS
Sodium channel conductance (*G*_*na*_)	1.445 kS
Leaked channel conductance (*G*_*l*_)	128 S

Finally, the required injection current was obtained to propagate an action potential in each axon of Figure 1B using a search method (bisection method) until the error was smaller than 10 μA. An action potential was elicited when the membrane potential was depolarized up to 80 mV in at least four neighboring nodes at successive times (Reilly, 2016). Then, the computational activation threshold of TI was the minimum required injection current among all the test axons. The total number of test axons was 80. This corresponds to a separation distance from each other of 0.1 mm (same to the mouse head model resolution). The axon with 0.1-mm resolution was sufficient to determine the “activation state region” (see Figure 1B). This is based on the assumption that the field distribution is rather smooth, and thus if a higher number of axons were considered, the “activation state region” does not change as the additional axons between the activated axons would be stimulated simultaneously and vice versa.

### Stimulation Scenarios

The stimulation condition is shown in Figure 1, which was defined following the mouse experiment in Grossman et al. (2017). To replicate the scenario in Grossman et al. (2017), the diameters of the circular anode and cathode were chosen as 1 and 5 mm, respectively, with a thickness of 0.5 mm. In addition, the electrodes comprised a sponge with saline solution. Two tACS circuits were modeled. Each anode was attached around the top of the brain, with its respective cathode attached to the side of the head (injection currents in the right and left hemispheres are defined as *I*_1_ and *I*_2_, respectively).

In this study, we fixed the total current injected into the two electrodes as 0.776 mA (*I*_1_+*I*_2_), which is the mean value reported in Grossman et al. (2017). The carrier frequencies of tACS were chosen as 1, 2, 3, and 4 kHz, and their differences were 5, 10, 15, 20, 50, and 100 Hz, as in Grossman et al. (2017).

**FIGURE 2:**
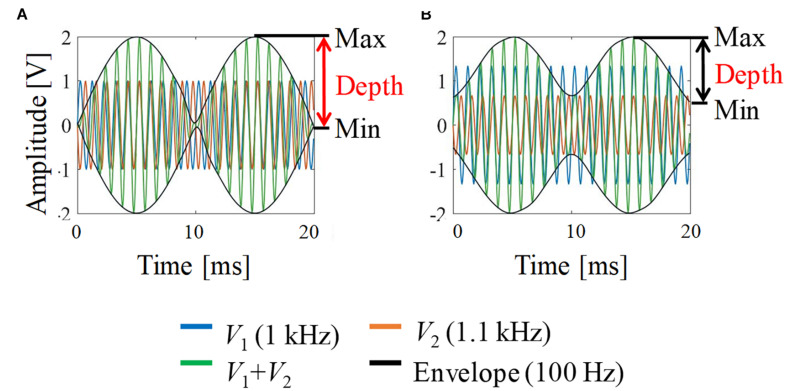
Envelope modulation waveforms. **(A)** *V*_1_ = *V*_2_; **(B)** *V*_1_:*V*_2_ = 2:1. For illustration, the carrier frequency was chosen as 1 kHz and the frequency difference (beat frequency) was 100 Hz (i.e., 1 and 1.1 kHz).

Figure 2 shows a schematic explanation and definition of typical envelop-modulated waveforms. It indicates the maximum, minimum, and depth of the envelope, which were used to quantify the neural activation in this study.

## Results

### Verification of Multiscale Model

The effect of the frequency difference (beat frequency) and carrier frequency of the two injection currents on the stimulation was evaluated computationally and compared with the motor threshold reported in a previous study (Grossman et al., 2017). The experimental motor threshold corresponded to activation of the right forepaw; thus, the target area was set to the expected motor area in the left hemisphere, where the thick nerves (corresponding to the pyramidal axon) were located (Neafsey et al., 1986). The computational activation threshold corresponded to the scenario using an amplitude ratio of *I*_1_ and *I*_2_ as the experiment.

**FIGURE 3:**
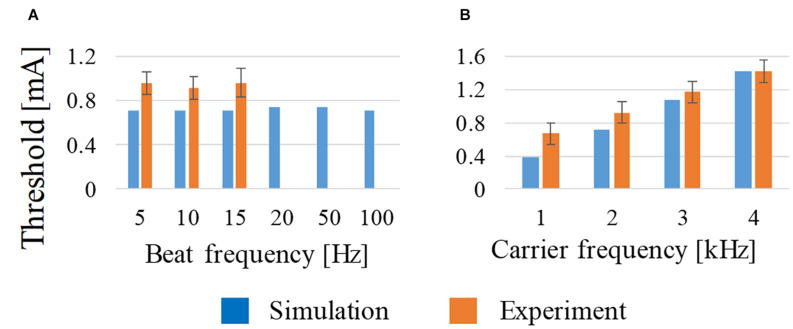
Effect on the activation threshold of **(A)** beat frequency at constant carrier frequency (2 kHz) and **(B)** carrier frequency at constant beat frequency (10 Hz) using amplitude ratio of *I*_1_ and *I*_2_ of 7:3. For comparison, experimental observations of behavioral stimulation are also shown.

As shown in Figure 3A, the beat frequency does not influence the activation threshold from 5 to 100 Hz, which is in good agreement with the measured threshold (Grossman et al., 2017). This tendency was computationally the same up to 100 Hz, although no measured results were reported above 15 Hz. From Figure 3B, the threshold of the stimulation increased with the frequency from 1 to 4 kHz, which is also in good agreement with the measured values.

### Effect of Amplitude Ratio on Stimulation Region

**FIGURE 4:**
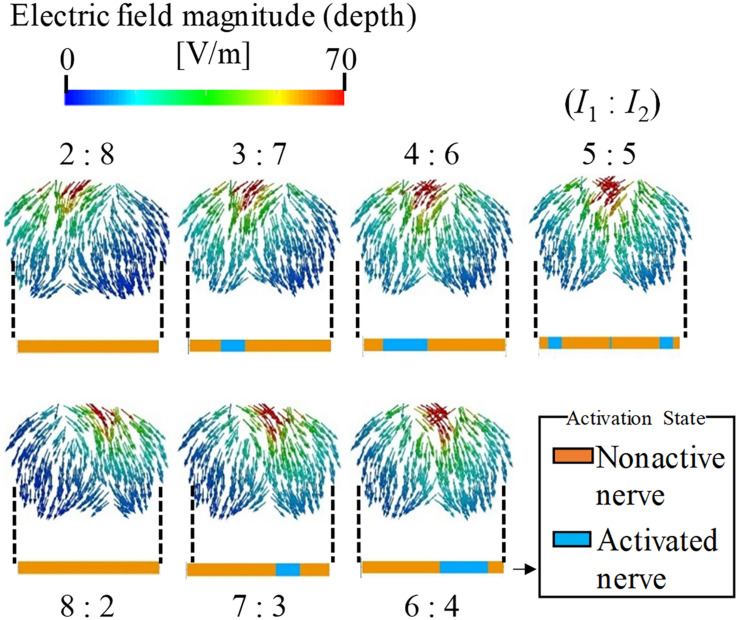
Stimulation regions of different stimulation conditions (*I*_1_:*I*_2_). The electric field vector direction was modified according to the injection current ratio (transverse plane). The magnitude is given by the envelope depth (see definition in Figure 2). Effects on neural activation are shown by the activation region that represents the state of the axon (active or non-active) for the different injection current ratios.

The effect of the amplitude ratio of the two injection currents on the stimulation was evaluated. Figure 4 shows the electric field direction (current direction) on the brain for different injection current ratios. The carrier and beat frequencies were 1 kHz and 10 Hz, respectively. As shown in this figure, the envelope depth of the electric field magnitude shifted in the direction opposite to that of the electrode, whose injection current amplitude was higher. Similarly, the multiscale model demonstrates that the activated neurons varied according to the amplitude ratios of the injection current, in which the activation changed in the same manner as the envelope depth. These results agree with the experimental results (Grossman et al., 2017).

### Metrics for Neural Stimulation Estimation

The different characteristics of the generated modulated signals of the electric field and electric potential were explored. Specifically, estimations of neuronal stimulation related to the maximum, minimum, and depth of the envelope were evaluated (see Figure 2).

**FIGURE 5:**
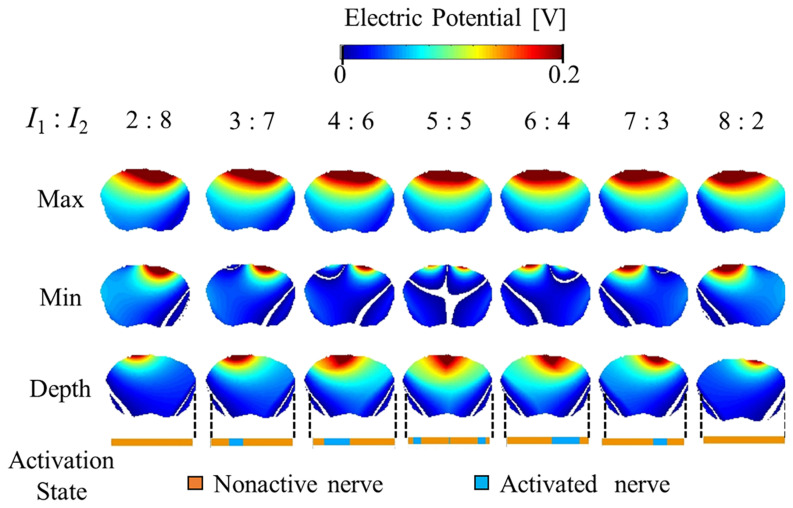
Electric potential distribution of the envelope for three metrics: maximum, minimum, and depth (see definitions in Figure 2). Activation state of the axons are indicated.

**FIGURE 6:**
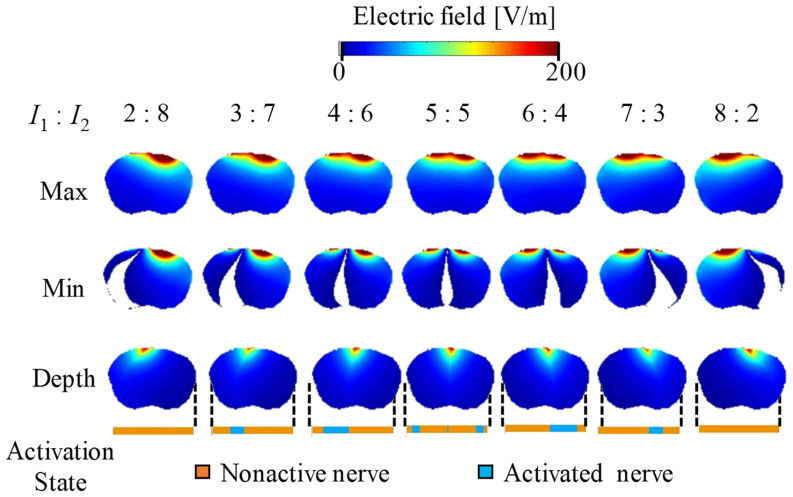
Electric field distribution of the envelope for three metrics: maximum, minimum, and (see definitions in Figure 2). Activation state of the axons is indicated.

Figures 5, 6 show the electric potential and electric field distributions, respectively. We observed that the region of the maximum value of the envelope (electric potential and electric field) cannot predict the region of the activated fibers. In contrast, the hotspot of the envelope depth agrees with the activated nerve region estimated using the multiscale model. In addition, the minimum value of the envelope (near zero) can be used to estimate the activated nerve region. In the case of depth, we noticed that the position of the hotspot using the electric potential envelope agreed with the center of the activated nerve region.

**FIGURE 7:**
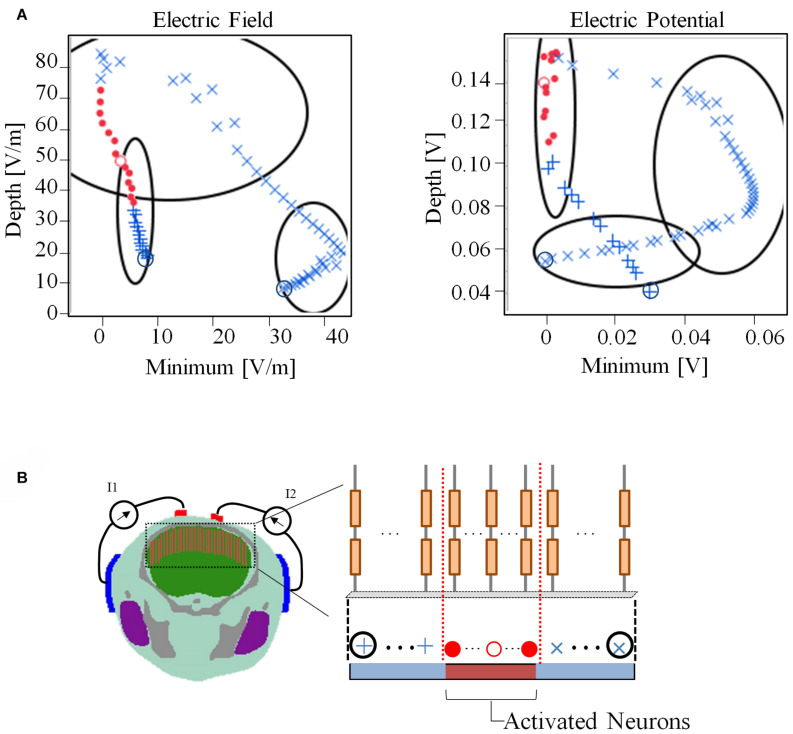
**(A)** Relationship between depth and minimum of the envelope for detection of activated neurons using electric field and electric potential. The regions have been separated into three groups based only on the depth and minimum values using hierarchical clustering. **(B)** The activation state (active or non-active) is coded in red and blue colors based on the multiscale model results. Consecutive neighboring nerve positions follow the path (+,^⋅^,×). The values of the electric field and electric potential are the averages along the nerve.

Figure 7 presents the relationship between the depth and minimum values of the envelope with the activated and non-activated nerves (case 7:3) based on multiscale model computations. In addition, the regions were separated into three groups based on hierarchical clustering. The computed activation region can be estimated based on the depth and minimum values of the electric potential envelope. Only considering the depth metric may determine the stimulation with the cost of including some non-active neurons next to the activation region. Whereas, only considering the minimum may include non-active neurons far from the activation region. Based on depth and minimum values of the electric field, the estimation group included more non-active neurons and more limited in the prediction.

## Discussion

In this study, we computationally investigated the effects of interference current patterns in a neural model embedded in a mouse brain using a multiscale model approach. To replicate the neuromodulation effects in the computational domain, we considered neural stimulation generated by interferential electric currents shaped by electrical and anatomical properties of the tissues in a realistic mouse head model for the first time. The model reproduced the experimental results by showing that that interferential stimulation acts on the neuronal axons. Also, this study showed which characteristics of the generated envelope can be used to predict the stimulation region to facilitate computational analysis intuitively.

Multiscale modeling was applied to replicate the experiments conducted in Grossman et al. (2017), the primary findings of which were: (i) beat frequency does not influence the threshold for the range considered, (ii) the threshold increases with an increase in the carrier frequency, and (iii) the stimulation region changes according to the ratio between the two injection currents. These were also reproduced in another study (Song et al., 2020). Our multiscale modeling replicated the three experiments for the first time using realistically shaped generated current effects on the neuronal model. Regarding the beat frequency effect, it is possible that the thresholds of the motor cortex may change at higher beat rates considering tetanic contraction of the muscle response. A larger current at a higher carrier frequency is necessary because a higher current amplitude is needed to overcome the low-pass filtering effect of the cell membrane. Furthermore, we computationally confirmed that the stimulated area can be controlled using the injected currents from two pairs of electrodes, which is one of the more important features of interferential stimulation to be exploited in brain stimulation techniques. Some differences between the computed and measured thresholds, as in Figure 3, are attributable to the positioning error in the electrode and/or fair modeling of the mouse brain (particularly for the cerebrospinal fluid), where magnetic resonance images may have insufficient resolution. Consequently, the nerve location was empirical.

We then explored the features of the generated envelope signal on the brain to determine which characteristics may serve as a physical metric of stimulation based on validated multiscale modeling. First, we observed that the maximum value of the generated electric field or electric potential envelope did not correspond to the exact activated nerve region given by the multiscale model. Instead, high values of the “depth” parameter (| maximum| − | minimum| values of the envelope) corresponded to better predictions of the activation region. We also noticed that the minimum value near zero (100% modulation) of the envelope may be used to determine the activated nerve region, which is the region in which both injection currents have the same intensity. Moreover, the combination of these metrics may help to better characterize the activated nerve region predicted using the multiscale model.

A limitation of our computational model is summarized as follows: first, the mouse model did not consider the cerebrospinal fluid, whose conductivity is higher than those of the remaining tissues, altering the current direction. However, the medical images, in general, do not warrant a resolution of less than 0.1 mm, and thus, unlike humans, tissue segmentation is insufficient. Therefore, the current flow due to the complexity of the brain might not be well represented. Also, neural trajectories have a radial orientation of neuronal paths from the cortex in the mouse (Wang et al., 2020). Our assumption of perpendicular axons for the sake of simplicity is more suitable for flat somatomotor cortical areas near the central part. Also, the effect of the axon curvature in the interior has a negligible effect as the stimulation occurs on the upper parts of the neuron (Gomez-Tames et al., 2020b).

In terms of validation where the experimental data is available (Grossman et al., 2017), the motor cortex was chosen. This area is suitable as it provides an easy marker of the stimulation effect to understand TI stimulation. Our findings can be extended to different brain areas, where the stimulation is characterized by axon stimulation. Also, the envelope formation at a specific part is expected to be useful to other brain regions. In addition, the cortical area is also a relevant target to investigate TI selectivity in the next study. For instance, generated scalp pain limits the intensity of injection currents in transcranial electrical stimulation (up to a few mA), producing weak electric fields in the brain. Instead, TI stimulation can selectively stimulate specific cortical parts while avoiding co-stimulation of peripheral scalp nerves. The next steps need to consider adding neural models not only in the cortical region but also in deep brain regions and peripheral nerves on the scalp (e.g., pain perception) to evaluate the reduction of co-stimulation.

In conclusion, we examined the effects of interferential stimulation on a neural model of the motor cortex. We considered the effects of the injection current on a realistic mouse head model. The results confirmed that varying the carrier frequency does not affect the threshold value, and the threshold value increases with an increase in the carrier frequency. The verified multiscale model for interferential stimulation was used to reveal the significant characteristics of the envelope generated by interferential stimulation to facilitate the estimation of the stimulation region. In particular, regions with a high modulation depth and low minimum envelope correspond to the activation region.

## Data Availability Statement

The raw data supporting the conclusions of this article will be made available by the authors, without undue reservation.

## Author Contributions

AH and JG-T conceived and designed the study. JG-T developed the interferential multiscale model. AA conducted the simulation experiments. AA and JG-T processed the data. All authors analyzed the data, wrote the manuscript, and read and approved the manuscript.

## Conflict of Interest

The authors declare that the research was conducted in the absence of any commercial or financial relationships that could be construed as a potential conflict of interest.
